# The lipidomic profile of the tumoral periprostatic adipose tissue reveals alterations in tumor cell’s metabolic crosstalk

**DOI:** 10.1186/s12916-022-02457-3

**Published:** 2022-08-18

**Authors:** Antonio Altuna-Coy, Xavier Ruiz-Plazas, Silvia Sánchez-Martin, Helena Ascaso-Til, Manuel Prados-Saavedra, Marta Alves-Santiago, Xana Bernal-Escoté, José Segarra-Tomás, Matilde R. Chacón

**Affiliations:** 1 Disease Biomarkers and Molecular Mechanisms Group, Institut d’Investigació Sanitària Pere Virgilii, Joan XXIII University Hospital, Universitat Rovira i Virgili, C/ Dr. Mallafré Guasch, 4. 43007, Tarragona, Spain; 2 Urology Unit, Joan XXIII University Hospital, Tarragona, Spain; 3 Pathology Unit, Joan XXIII University Hospital, Tarragona, Spain

**Keywords:** Periprostatic adipose tissue, Lipidomic, Lipid metabolism, De novo fatty acid synthesis, Prostate cancer

## Abstract

**Background:**

Periprostatic adipose tissue (PPAT) plays a role in prostate cancer (PCa) progression. PPAT lipidomic composition study may allow us to understand the tumor metabolic microenvironment and provide new stratification factors.

**Methods:**

We used ultra-high-performance liquid chromatography-mass spectrometry-based non-targeted lipidomics to profile lipids in the PPAT of 40 patients with PCa (*n* = 20 with low-risk and *n* = 20 high-risk). Partial least squares-discriminant analysis (PLS-DA) and variable importance in projection (VIP) analysis were used to identify the most relevant features of PPAT between low- and high-risk PCa, and metabolite set enrichment analysis was used to detect disrupted metabolic pathways. Metabolic crosstalk between PPAT and PCa cell lines (PC-3 and LNCaP) was studied using ex vivo experiments. Lipid uptake and lipid accumulation were measured. Lipid metabolic-related genes (*SREBP1*, *FASN*, *ACACA*, *LIPE*,* PPARG*,* CD36*,* PNPLA2*,* FABP4*,* CPT1A*,* FATP5*,* ADIPOQ*), inflammatory markers (*IL-6*, *IL-1B*, *TNFα*), and tumor-related markers (*ESRRA*, *MMP-9*, *TWIST1*) were measured by RT-qPCR.

**Results:**

Significant differences in the content of 67 lipid species were identified in PPAT samples between high- and low-risk PCa. PLS-DA and VIP analyses revealed a discriminating lipidomic panel between low- and high-risk PCa, suggesting the occurrence of disordered lipid metabolism in patients related to PCa aggressiveness. Functional analysis revealed that alterations in fatty acid biosynthesis, linoleic acid metabolism, and β-oxidation of very long-chain fatty acids had the greatest impact in the PPAT lipidome. Gene analyses of PPAT samples demonstrated that the expression of genes associated with de novo fatty acid synthesis such as *FASN* and *ACACA* were significantly lower in PPAT from high-risk PCa than in low-risk counterparts. This was accompanied by the overexpression of inflammatory markers (*IL-6*,* IL-1B*, and *TNFα*). Co-culture of PPAT explants with PCa cell lines revealed a reduced gene expression of lipid metabolic-related genes (*CD36*, *FASN*, *PPARG*, and *CPT1A*), contrary to that observed in co-cultured PCa cell lines. This was followed by an increase in lipid uptake and lipid accumulation in PCa cells. Tumor-related genes were increased in co-cultured PCa cell lines.

**Conclusions:**

Disturbances in PPAT lipid metabolism of patients with high-risk PCa are associated with tumor cell metabolic changes.

**Supplementary Information:**

The online version contains supplementary material available at 10.1186/s12916-022-02457-3.

## Background

Periprostatic adipose tissue (PPAT) is a specific fat depot that surrounds the prostate and may constitute an important active component in the metabolic tumor interplay, adapting to the metabolic needs of prostate cancer (PCa) cells, thus participating in tumorigenesis and resistance to treatments [1].

Lipid metabolism has been found to be important in PCa [2]. Fatty acid (FA) biosynthesis is dysregulated in PCa [3]. Tumor cells can establish bidirectional crosstalk with adipose tissue, inducing lipolysis and FA release that can be subsequently stored and used by tumor cells [4]. Indeed, cell co-culture studies have shown that PCa cell lines incorporate FA from neighboring isolated adipocytes [5]. Because PCa cells can induce adipocyte lipolysis to increase their intake of FA [6], suppressing FA uptake might be an interesting strategic point for designing future therapies in patients with localized PCa tumors.

Detailed and quantitative studies of the PPAT lipidome are limited and have been focused on fatty acids (FAs), considered important determinants of the lipid composition of adipose tissue [7–10]. PPAT FA profile analysis reported few significant differences only according to the ethno-geographical origin [8], particularly when comparing Caucasian with African-Caribbean where aggressiveness was associated with lower levels of linoleic acid. Others found differences in FA composition when comparing PPAT samples with benign prostate hyperplasia, with higher levels of palmitic in PPAT tissue [7]. Interestingly, a study analyzing basal secreted FA profile from PPAT explants revealed no differences in relation to tumor aggressiveness when patients were dichotomized by Gleason score into less or more aggressive PCa [9].

A deeper characterization of PPAT may lead to a better understanding of the disease and possibly allow new stratification factors. Therefore, in the present study, we aimed to analyze the lipidome of PPAT using an untargeted lipidomic approach. We obtained PPAT samples from patients undergoing radical prostatectomy in an attempt to identify lipid signatures associated with PCa aggressiveness. Additionally, we performed metabolite enrichment and gene expression analysis to identify biologically meaningful patterns and to search for possible correlations between PPAT lipidomic metabolites and metabolic-related genes. Finally, we performed co-culture experiments to question lipid microenvironment crosstalk.

## Methods

### Patients and tissues

A total of 40 PPAT samples were obtained during surgical procedures in patients with PCa consecutively treated by radical prostatectomy at the University Hospital Joan XXIII, Tarragona (Spain). The surgery was performed laparoscopically assisted by a *Da Vinci* robot. Once the anterior surface of the prostate had been surgically exposed, the surrounding PPAT surface was removed and 1–2 g of this fat tissue was immediately placed in a sterile container, washed twice in PBS, and stored in vapor phase of liquid nitrogen.

All patients signed an informed consent. Clinical characteristics are summarized in Additional file 1. Patients were stratified based on the International Society of Urological Pathology (ISUP) consensus conference on Gleason grading of prostatic carcinoma [11] as high-risk (III, IV, and V) (*n* = 20) and low-risk (I and II) (*n* = 20). The study was performed according to the provisions of the Declaration of Helsinki and was approved by the local ethics committee and adhered to current legal regulations (Biomedical Research Law 14/2007, Royal Decree of Biobanks 1716/2011, Organic Law15/1999 of September 13 Protection of Personal Data). All methods were approved and performed in accordance with guidelines and regulations of the Ethical Committee for Clinical Research (CEIM) from the *Pere Virgili* Research Institute (CEIM205/2020).

### Metabolomics multiplatform

To characterize adipose tissue metabolic profiles, FA methyl ester (FAME), acylcarnitine, and lipidomic analysis was carried out using gas chromatography-mass spectrometry (GC–MS/MS), liquid chromatography-mass spectrometry (LC–MS/MS), and liquid chromatography quadrupole time-of-flight mass spectrometry (LC-QTOF-MS), respectively. For lipidomic analysis, two different LC‐QTOF‐MS‐based platforms were used to analyze methanolic tissue extracts (LIP-I) and chloroform/methanol tissue extracts (LIP-II). The methanol extract platform included free FA, bile acids, steroids, oxylipins, and lysophospholipids (LP). The chloroform/methanol extract platform provided coverage over sphingolipids (SM), monoacylglycerols (MG), diacylglycerols (DG), triacylglycerols (TG), phosphatidylcholines (PC), and ether-phosphatidylcholines (PC-O).

Total FAs were derived from the quantitative analysis of FAME. The acylcarnitine and lipidomic characterizations were performed using internal standards to correct the response of each detected compound based on their family similarity, providing a semi-quantitation as an internal standard response ratio. Specific extraction protocols and mass spectrometry-based analyses are detailed in Additional file 2: Additional Material and Methods.

The number of metabolites obtained by the three different techniques was as follows: LIP-I, *n* = 120 lipids; LIP-II, *n* = 122 lipids; and FAME, *n* = 22.

### *Ex vivo *co-culture of PPAT explants and PCa cell lines

We designed Transwell co-culture assays of PPAT explants (7 low-risk and 3 high-risk) with 2 PCa cell lines. The prostate cancer cell lines PC-3 (androgen-insensitive) and LNCaP (androgen-sensitive) were purchased from Sigma-Aldrich (Barcelona, Spain). PC-3 cells were cultured in Ham’s F-12 K (Kaighn’s) Medium (1:1 mixture) with L-glutamate (Invitrogen/Gibco, Fisher Scientific SL, Madrid, Spain). LNCaP cells were cultured in RPMI 1640 medium (Merck KGaA, Darmstadt, Germany) supplemented with 1 mM sodium pyruvate (Gibco). PC-3 and LNCaP cultures were supplemented with 10% fetal bovine serum, 1% penicillin/streptomycin, and 5 μg/mL Plasmocin® (Invivogen, IBIAN Technologies, Zaragoza, Spain). Cells were seeded into Transwell 0.4-μm pore size cell culture inserts (Fisher scientific, Barcelona, Spain) at 50,000 cells/0.9 cm^2^ in the same medium at 37 °C and 5% CO_2_ during 24 h. The next day, the medium was exchanged for serum-free medium, for 24 h. Subsequently, 50 mg of fresh PPAT was washed with PBS twice and added to the lower Transwell chamber in 1 mL of M199 medium with 10% FBS and 1% penicillin/streptomycin in 25 mM HEPES. Each sample was tested in duplicate. The co-culture was maintained at 37 °C and 5% CO_2_ for 48 h in the same medium. Subsequently, cells and tissue explants were removed, and RNA was extracted.

### RNA extraction and real-time gene expression

Total RNA was extracted from 50 mg of either frozen PPAT or fresh PPAT explants (after co-culture) using the RNeasy Lipid Tissue Mini Kit (Qiagen, Germantown, MD). PC-3 and LNCaP RNA was extracted using the RNeasy Mini Kit (Qiagen). Total RNA was quantified by absorbance measurement, and its purity was assessed by the OD260/OD280 ratio. RNA was retrotranscribed to cDNA using a High Capacity cDNA-to-RNA Kit (Applied Biosystems, Foster City, CA) and the following genes were tested using Taqman assays (Applied Biosystems): *SREBP1* (hs01088691_m1), *PPARG* (hs00234592_m1), *FASN* (hs01005622_m1), *ACACA* (hs01046047_m1), *LIPE* (hs00193510_m1), *IL-6* (hs00985639_m1), *IL-1B* (hs01555410_m1), *TNFα* (hs99999043_m1), *CD36* (hs00169627_m1), *FABP4* (hs00609791_m1), *CPT1A* (hs00912671_m1), *PNPLA2* (hs00386101_m1), *FATP5* (hs00202073_m1), *ADIPOQ* (hs00605917_m1), *ESRRA* (hs01067166_g1), *TWIST1* (hs00361186_m1), and *MMP-9* (hs00957662_m1). The value for each sample was normalized to the expression of GAPDH. SDS software 2.3 and RQ Manager 1.2 (Applied Biosystems) were used to analyze the results with the comparative Ct method (2^−∆∆Ct^). To compare low- versus high-risk, PPAT data were expressed as an *n*-fold difference relative to a calibrator (a mix of 1 sample of PPAT and 1 sample of abdominal adipose tissue from our Biobanc collection). Co-culture cell lines and co-culture explant PPAT data were expressed as an *n*-fold difference relative to control.

### Lipid uptake and lipid accumulation assays

PCa cells were seeded in 96-well clear flat-bottom black plates (Thermo Fisher Scientific, Barcelona, Spain) and co-cultured with 10 mg of PPAT for 48 h. To quantify fatty acid uptake, the growth medium was exchanged with TF2-C12 fatty acid (Sigma-Aldrich), and cells were incubated at 37 °C for 30 and 60 min. Cellular uptake was measured on Varioskan Lux Reader (Thermo Fisher Scientific) by measurement of fluorescence intensity (*λ* exposition = 485/*λ* emission = 515 nm). Lipid content was measured with Nile Red (Sigma-Aldrich). Briefly, media were removed, cells were washed twice with PBS, and lipids were stained with 1.1 mg/mL Nile Red for 15 min at 37 °C. After incubation, cells were washed with PBS followed by measurement of fluorescence intensity (*λ* exposition = 488/*λ* emission = 590 nm).

### Statistical analysis

For the PPAT study, the sample size was calculated using G*Power 3.1.9.7 (https://www.psychologie.hhu.de/arbeitsgruppen/allgemeine-psychologie-und-arbeitspsychologie [12]. Assuming a change of twofold between groups and similar group variances, with an average power > 80% and a false discovery rate of 5%, a minimum of 17 patients were needed in each group. Variables not normally distributed are presented as medians and interquartile ranges and were compared with the Mann–Whitney *U* test. Multivariate stepwise backward regression analysis was employed in order to evaluate the independent predictors associated with PCa aggressiveness. Statistical analyses were performed using the Statistical Package for the Social Sciences, version 22 (SPSS, Chicago, IL). The R software (https://cran.r-project.org/web/packages/Information/index.html) was downloaded. To achieve the best discriminatory model between studied groups, we performed variable importance in projection (VIP) score analysis and partial least squares-discriminant analysis (PLS-DA) using *MetaboAnalystR* package (https://github.com/xia-lab/MetaboAnalystR). Pathway enrichment was performed by using MetaboAnalyst 5.0 software (https://www.metaboanalyst.ca), and we selected the metabolite set library from SMPDB (The Small Molecule Pathway Database). GraphPad Prism 7.0 was used for the box plot analysis of the different metabolites. For ex vivo experiments, at least 6 co-culture experiments were performed with different human PPAT explant tissues and 2 PCa cell lines. Statistical significance was evaluated with Mann–Whitney tests. Results with *p* < 0.05 were considered statistically significant.

## Results

### Lipid profiling of PPAT

We performed untargeted lipidomic profiling of 40 PPAT samples using LC–MS/MS and GC–MS/MS platforms. Patients were stratified according to ISUP GG into two categories: low-risk (ISUP groups I and II, *n* = 20 tissues) and high-risk (ISUP groups III, IV, and V, *n* = 20 tissues). Patients’ characteristics are summarized in Additional file 1. Of 264 individual lipid metabolites detected, 67 were significantly differentially expressed between low- and high-risk PPAT samples (Additional file 3).

**Fig. 1:**
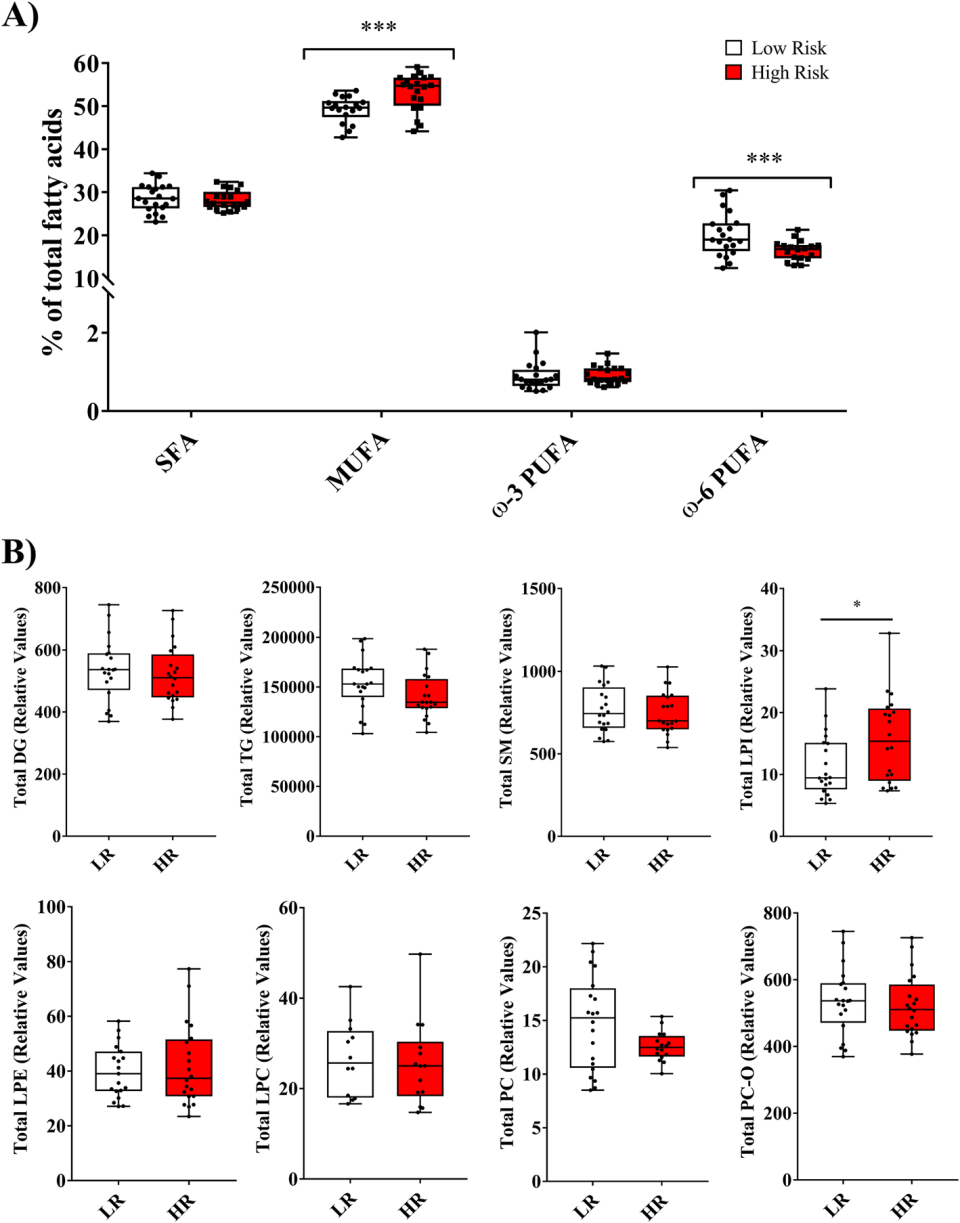
Identified lipid species in PPAT samples. **A** Total fatty acids. Error bars represent standard deviation (SD) of the mean. ***p < 0.001. **B** Other annotated lipids divided by lipid category. Box shows the median, quartiles, and extreme values. *p < 0.05. Abbreviations: SFA, saturated fatty acid; MUFA, monounsaturated fatty acids; ω-3 PUFA, omega 3 polyunsaturated fatty acids; ω-6 PUFA, omega 6 polyunsaturated fatty acids; DG, diglyceride; TG, triglyceride; SM, sphingomyelin; LPI, lysophosphatidylinositol; LPE, lysophosphatidylethanolamine; LPC, lysophosphatidylcholine; PC, phosphatidylcholine (diacylglycerol); PC-O, phosphatidylcholine (alkyl-acyl-glycerol)

Analysis of total FA profile obtained after FAME analysis (reveals all FA cellular composition, including acyl chains and free fatty acids) showed that the percentage of total saturated FA (SFA) was not different between the low- and high-risk groups whereas a higher percentage of monounsaturated FA (MUFA) was observed in high-risk PPAT tissue when compared with low-risk counterpart’s (27.59% vs 28.53%, respectively; *p* = 0.001). Analysis of polyunsaturated FA (PUFA) revealed a significant difference for ω-6 PUFA, which were lower in abundance in high-risk PPAT samples compared with low-risk (17.05% vs 18.98% respectively, *p* = 0.001) (Fig. 1A, Additional file 4).

No changes were observed between the two studied groups for the following metabolites obtained by LIP-II analysis: DG, *p* = 0.565; SM, *p* = 0.565; lysophosphatidylethanolamine (LPE), *p* > 0.9; LPC, *p* = 0.873; PC, *p* = 0.155; and PC-O, *p* = 0.337. By contrast, a significant difference (*p* < 0.05) was detected in the total levels of lysophosphatidylinositol (LPI), which were higher in high-risk PPAT samples. Also, a trend for lower levels of TG (*p* = 0.063) was found in high-risk PPAT samples (Fig. 1B).

### Analysis of PPAT lipid signatures for prognosis of PCa

To identify the most relevant lipid features that would allow us to correctly stratify low-risk versus high-risk patients based on the PPAT lipid profile, we performed PLS-DA on a data set of 70 variables (including the 67 significantly deregulated lipid metabolites shown in Additional file 3 and we also included the variables total SFA, total MUFA, and total PUFA). The score plot of the PLS-DA model showed a separation between patients regarding PCa aggressiveness (Additional file 5: Fig. S1A). We then performed VIP analysis to examine the contribution of the 70 variables in determining the degree of PCa aggressiveness, finding that 33/70 variables had a VIP score ≥ 1 and were therefore considered as important in the model for determining PCa aggressiveness (Additional file 5: Fig. S1B).

**Fig. 2:**
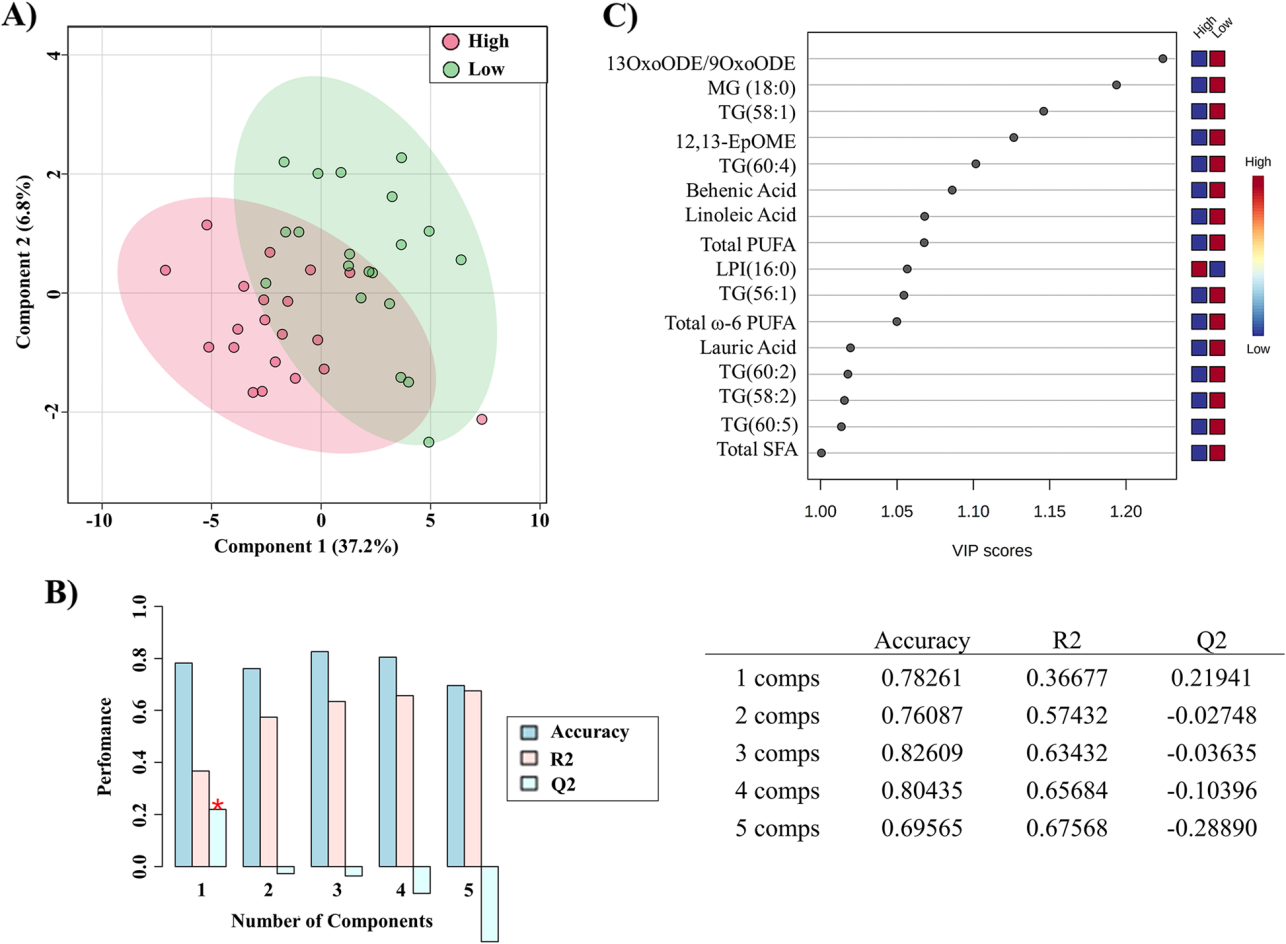
Lipid analysis of PPAT. **A** Principal component analysis score plot of the 16 selected signatures. **B** Variance explanation (%) for each principal component is indicated. **C** Variable Importance of projection (VIP) analysis of the selected lipid signatures with VIP scores ≥ 1. Abbreviations: 13-oxoODE/9oxoODE, 13-Oxo-9,11-octadecadienoic acid/9-oxo-10E,12Z-octadecadienoic acid; 12,13-EpOME, vernolic acid; MG, monoglyceride, TG, triglyceride; LPI, lysophosphatidylinositol; ω-6 PUFA, omega 6 polyunsaturated fatty acids; SFA, saturated fatty acid

**Fig. 3:**
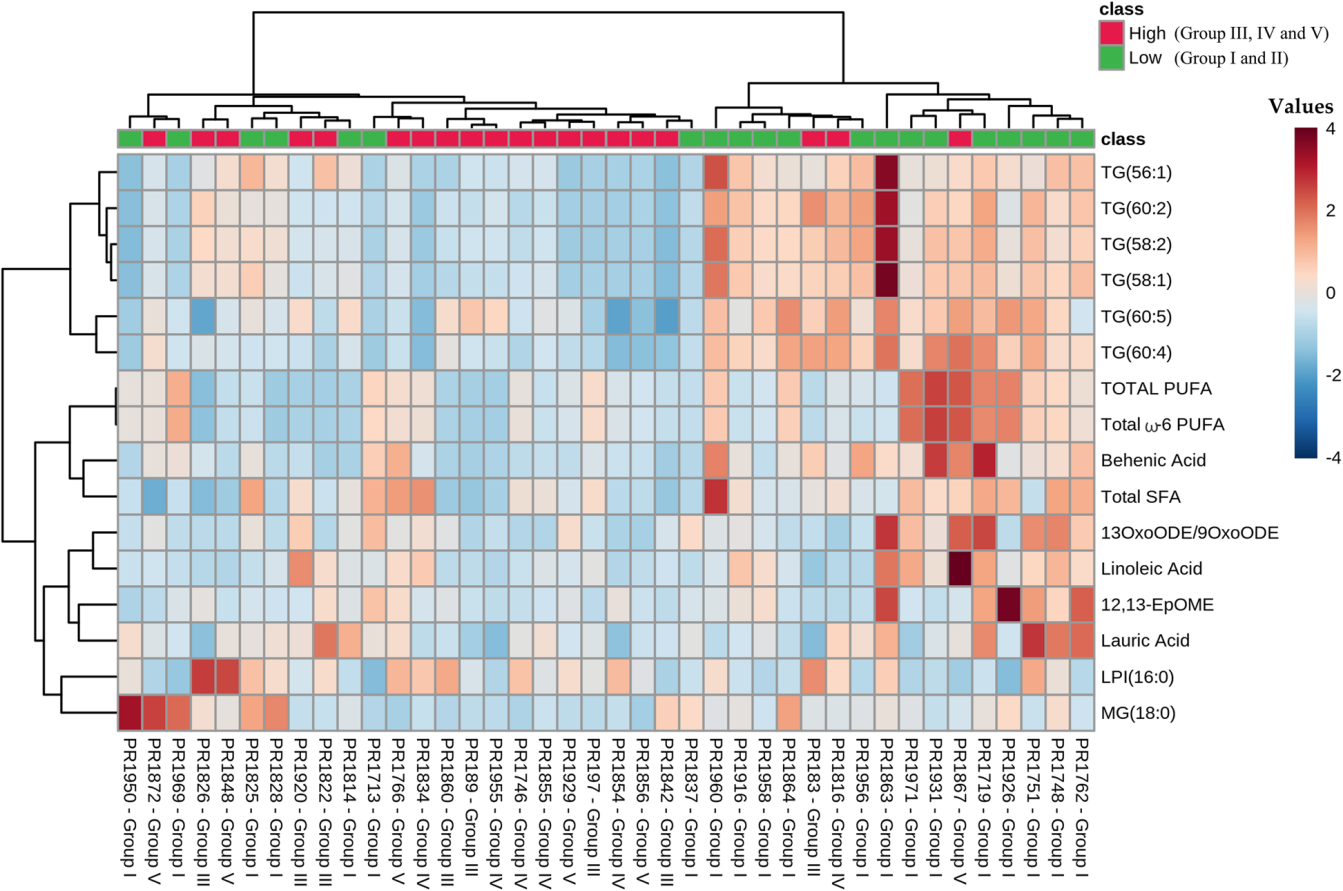
Heat map of the clustering analysis. Samples of PPAT categorized by ISUP group naturally clustered into experimental studied groups: low-risk (group I) and high-risk (groups III, IV, and V). Metabolite intensities are displayed as colors ranging from red grading (more abundant metabolites) to blue grading (less abundant metabolites). Classification of lipid according to extraction method: FAME: ω-6 PUFA, SFA, total PUFA, and behenic acid. LIP-I: 13-OxoODE/9oxoODE; 12,13-EpOME; LPI, lauric acid and linoleic acid. LIP-II: MG, and TG

The selected 33 variables were then back evaluated with PLS-DA to test the strength of the model, and again the patients were segregated into two differentiated groups (Fig. 2A). The PLS-DA model over fitting, measured as the Q2/R2 ratio (R2—how well the model predicts the calibration of variables, and Q2—how well the model predicts PCa aggressivity) was 0.59, indicating that the model fitted well (Fig. 2B). A model is considered predictive when the Q2/R2 ratio is greater than 0.5 [13]. To obtain the minimum number of significant lipidomic PPAT signatures that could separate the low- and high-risk PCa groups, we performed a second VIP analysis and the model showed that only 16 of the 33 variables had a VIP score ≥ 1 (Fig. 2C). The heat map in Fig. 3 shows that based on these 16 features the PPAT samples, categorized by ISUP grade group, naturally clustered into 2 separate groups corresponding to low- and high-risk PCa.

Multivariate stepwise backward regression analysis with the 16 signatures from VIP panel allowed us to evaluate the independent lipid metabolite predictors associated with PCa aggressiveness. Results showed that 12,13-EpOME (*B* = 0.008, *p* = 0.039, 95% CI = 0–0.779) and MG (18:0) (*B* = 0.942, *p* = 0.005, 95% CI = 0.9–0.982) were independently associated with PCa aggressiveness.

### Lipid derangements in PPAT from patients with PCa are related to metabolic alterations

**Fig. 4:**
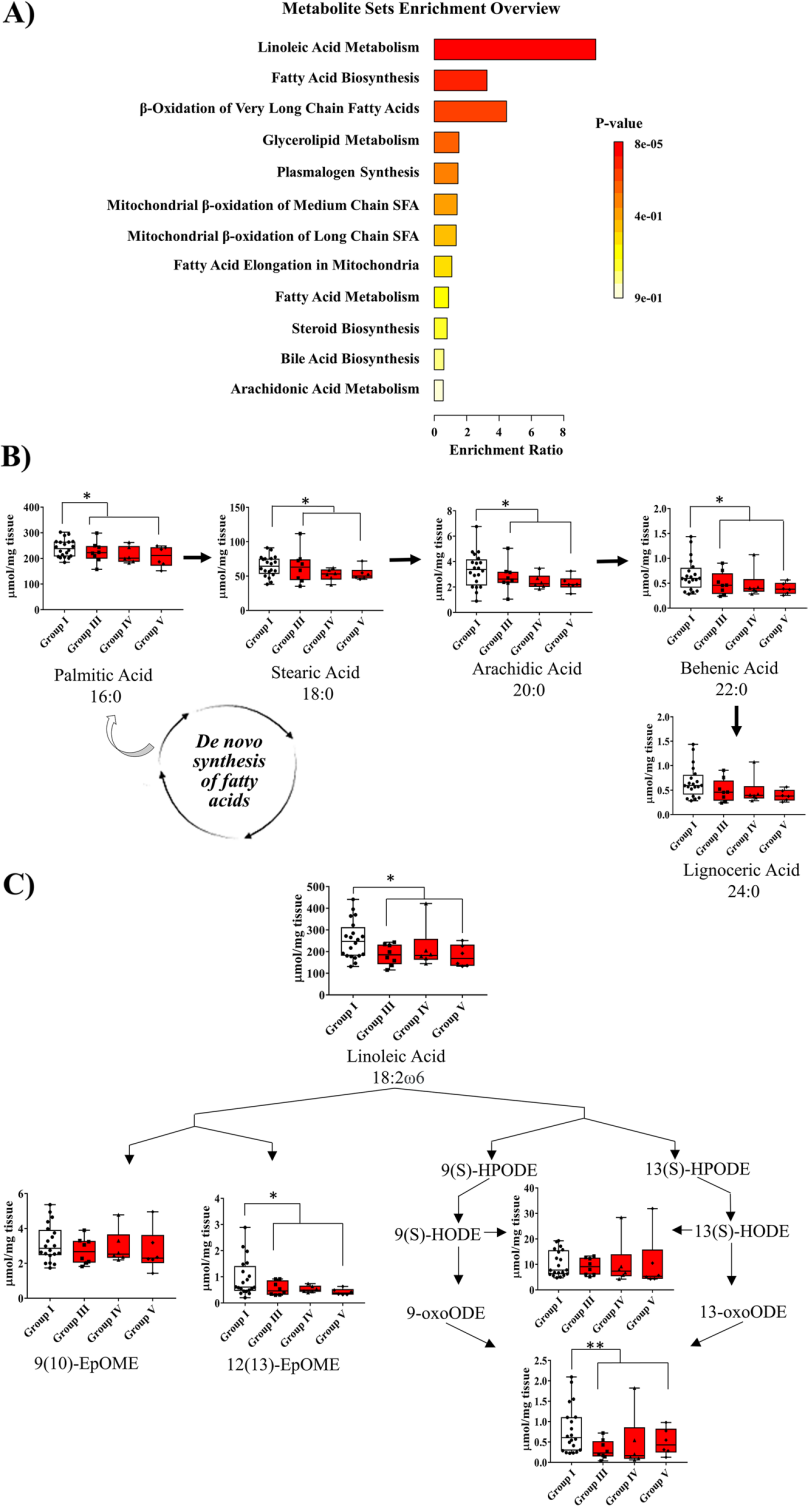
Biochemical metabolic analysis. **A** Metabolic set enrichment analysis showing altered lipid signatures analyzed by SMPDB metabolite set library. **B**, **C** Significantly altered lipid metabolite box plots allocated to related pathways. Patients were stratified regarding ISUP group. Statistic comparation was performed between high-risk (groups III, IV, and V) and low-risk (group I). Each box shows the median, quartiles, and extreme values. *p < 0.05, **p < 0.01

The PPAT lipid signatures revealed an apparent disorder in lipid metabolism according to PCa pathogenesis. To obtain a global overview of the altered metabolic pathways, we performed metabolite set enrichment analysis using MetaboAnalyst 5.0 and SMPDB metabolite set library with the all-lipid metabolites outlined in Fig. 3. These functional approaches revealed that alterations in linoleic acid metabolism, biosynthesis of FA, and β-oxidation of very long-chain fatty acid had the highest impact in the PPAT lipidome (Fig. 4A) (*p* < 0.05). Then, several SFA metabolites’ profiles obtained by FAME (Additional file 4) were mapped onto de novo lipid synthesis pathways, and we observed that the amount of palmitic acid and the total amount of its intermediate products, which may be further elongated to form other FA, showed a gradually decreasing trend when patients were stratified by ISUP group. When samples were grouped into low- and high-risk PPAT, significant differences were observed in the amounts of palmitic acid, stearic acid, arachidic acid, and behenic acid (Fig. 4B).

When mapping metabolites related to the linoleic acid pathway, we observed that in general, metabolites showed a gradual decrease when PPAT samples were stratified by ISUP group (Fig. 4C). Significant lower amounts of linoleic acid (LA) only appeared in high-risk PPAT samples when grouped by lower and high-risk PPAT (Fig. 4C). Linoleic acid can be oxygenated by 15-lipoxygenase 1 (LOX-1) in humans, primarily to 13-oxoODE or 9-oxoODE, which were also found to be significantly lower in high-risk than in low-risk PPAT.

Linoleic acid can also be converted by cytochrome P450 to epoxy-octadecenoic acids (EpOMEs) in the form of either 9(10)-EpOME (leukotoxin, coronaric acid) or its regioisomer 12(13)-EpOME (isoleukotoxin, vernolic acid); both metabolites were also lower in abundance in high-risk PPAT than in low-risk PPAT (Fig. 4C).

### PPAT from aggressive PCa tumors exhibits an altered gene expression profile related to lipid metabolism and inflammation

**Fig. 5:**
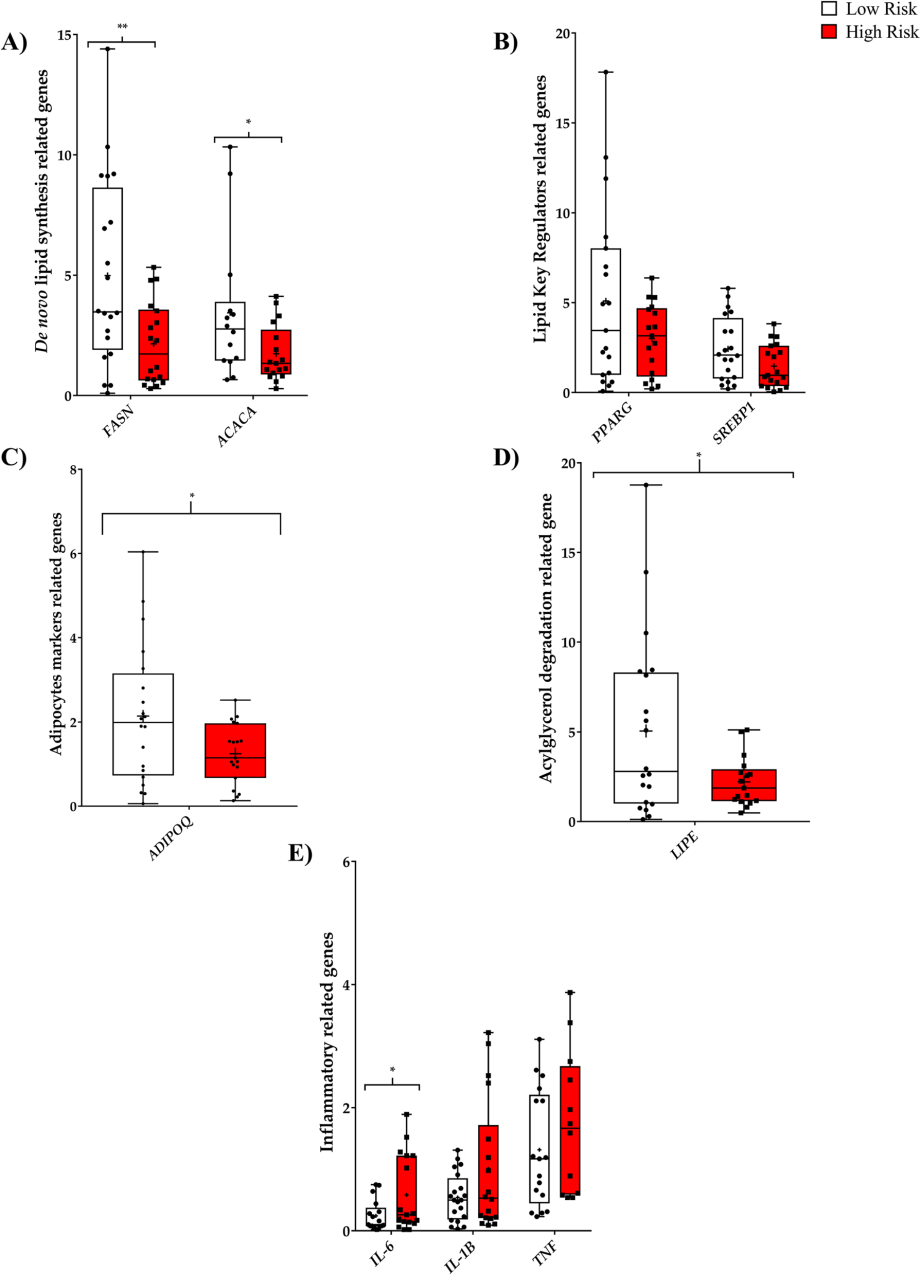
Analysis of gene expression of PPAT samples. **A–D** PPAT displays an altered expression profile of lipid metabolism-related genes and **E** inflammatory-related genes. Each box shows the median, quartiles, and extreme values of *n* = 20 samples

We next aimed to determine the specific contribution of selected genes in relation to the altered metabolic pathways. We evaluated the expression of sterol regulatory element binding transcription factor 1 (*SREBP1*), fatty acid synthase (*FASN*), and acetyl-CoA carboxylase (*ACACA*) genes, which are involved in de novo FA synthesis. Levels of *FASN* and *ACACA* were significantly lower in high-risk PPAT (Fig. 5A), whereas levels of *SREBP1* were reduced in high-risk PPAT when compared with low-risk PPAT (Fig. 5B) but did not reach significance. All this data indicates a diminished de novo FA synthesis, in good agreement with the levels of these metabolite pathway products in PPAT (Fig. 4).

Linoleic acid can be converted to 13-OxoODE, which is an endogenous ligand for peroxisome proliferator-activated receptor gamma (*PPARG*) [14]. When we measured *PPARG* expression levels, we observed a downregulating trend in high-risk PPAT when compared with low-risk PPAT samples (Fig. 5B). This finding can be related to the lower levels of 13-oxoODE in high-risk PPAT (Fig. 4C). A significant reduction in *ADIPOQ* gene expression, a gene directly regulated by PPARG, was observed in high-risk PPAT samples when compared with their low-risk counterparts (Fig. 5C).

We also observed that the expression of hormone-sensitive lipase (*LIPE*) was significantly lower in high-risk PPAT samples than in low-risk samples (Fig. 5D), which has also been described in breast cancer-associated adipocytes [4].

Interestingly, we also revealed an altered inflammatory state in high-risk PPAT samples, demonstrated predominantly by the significantly higher expression of the proinflammatory cytokine interleukin 6 (*IL-6*) in addition to a trend for higher levels of interleukin 1 (*IL-1B*) and tumor necrosis factor alpha (*TNFα*) (Fig. 5E).

### *Ex vivo *co-culture of PPAT explants with PCa cell lines triggers changes in the expression of lipid-, inflammatory-, and tumor-related genes

The observed reduction in metabolites from pathways associated with de novo FA synthesis and the increased inflammatory profile led us to question if these gene expression alterations were due to direct contact with tumor cells.

**Fig. 6:**
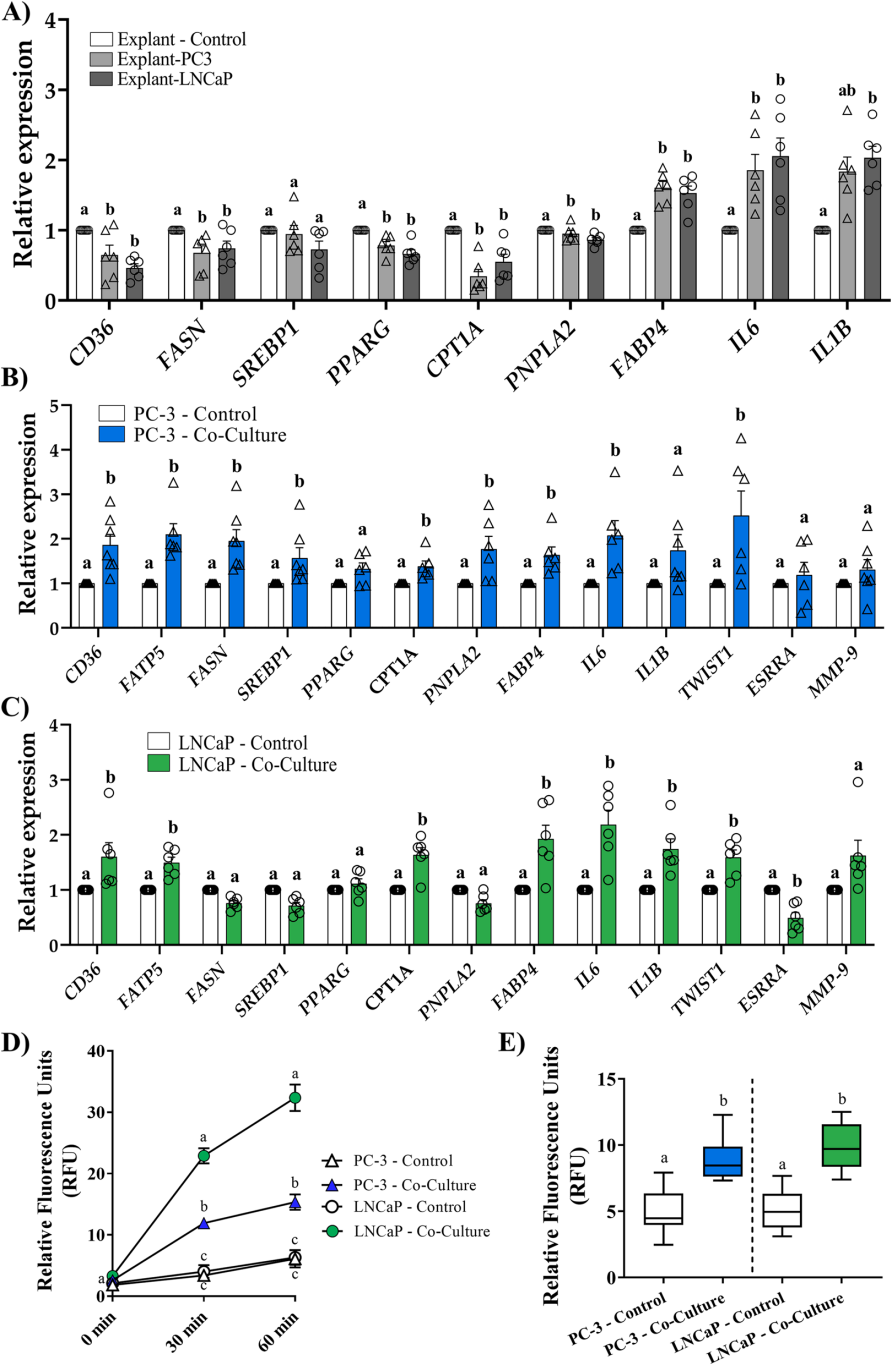
A bidirectional crosstalk occurs between PPAT explants and PCa cell lines. PCa cell lines (PC-3 or LNCaP) were co-cultivated with PPAT explants for 48 h and expression of lipid-, inflammatory-, and tumor-related genes were evaluated by RT-qPCR. **A** Co-cultured explants with LNCaP or PC-3 cells. **B** Co-cultured PC-3 cells. **C** Co-cultured LNCaP cells. **D** Uptake of the fatty acid analog TF2-C12 at 30 and 60 min in PCa cell lines co-cultured with PPAT explants. **E** Lipid accumulation measured with Nile Red dye after 15-min incubation in PCa cell lines co-cultured with PPAT explants. Different lettering over the boxes indicates statistical differences. Significant differences are established at *p* < 0.05. Data are expressed as mean ± SEM (*n* = 6 experiments)

To address this, we co-cultured PPAT explants with 2 different PCa cell lines (PC-3 and LNCaP) and then performed gene expression analysis. Results showed that regardless of the cell line used in the co-culture experiment, the expression of membrane lipid transporter *CD36* and the expression of genes implicated in de novo lipogenesis such as *FASN* and *PPARG*, a transcription factor implicated in lipid metabolism and inflammation, were all significantly downregulated in PPAT explants (Fig. 6A). Of note, the key gene regulator factor *SREBP1* was slightly reduced but did not reach significance (Fig. 6A). The expression of carnitine palmitoyltransferase 1A (*CPT1A*), responsible for the translocation of FA from the cytosol to the mitochondrial matrix, was also reduced. By contrast, the inflammatory profile in PPAT co-cultured explants was elevated, as shown by an upregulation of cytokines such as *IL-6* and *IL-1B*. Interestingly, fatty acid binding protein 4 (*FAPB4*) expression was higher after co-culture, indicating an active cytoplasmatic lipid mobilization process between PPAT and PCa cell lines (Fig. 6A). The expression of the lipase *PNPLA2* was reduced in PPAT explants after co-culture, indicating that lipolysis was likely not upregulated under this culturing condition (Fig. 6A). Of note, no differences in co-cultured explants between high-risk and low-risk tumors were observed following co-culture with PCa cell lines in the lipid and inflammatory genes here analyzed (Additional file 6: Fig. S2A-S2B).

Conversely, co-cultured PCa cell lines (both PC-3 and LNCaP) showed a significant increase in the expression of lipid metabolism-related genes such as *CD36*, *FATP5*, *CPT1A*, and *FABP4* and of the inflammation-related genes *IL-6* and *IL-1B* (Fig. 6B, C). Noteworthy, while *FASN*, *SREBP1*, *PPARG*, and *PNPLA2* were upregulated in PC-3 after PPAT explant co-culture (Fig. 6B), no changes were observed in these genes in LNCaP cells after co-culture (Fig. 6C).

Alteration in PCa cell aggressiveness was also observed in both cell lines after PPAT explant co-culture, demonstrated by a significant increase in expression levels of genes implicated in tumor cell proliferation (*ESRRA*) and in tumor invasive and metastatic potential (*MMP-9*, *TWIST1*) (Fig. 6B, C).

An increase in fatty acid uptake is shown in both cell lines (Fig. 6D) after explant co-culture. Also, both PCa cell lines exhibited enhanced lipid accumulation after explant co-culture (Fig. 6E). Of note, LNCaP co-culture cells showed a significantly higher uptake when compared with co-culture PC-3 cells. No differences in accumulation or in uptake rates were observed in the PCa cell lines studied (PC-3 and LNCaP) regardless of PPAT explant aggressiveness (Additional file 6: Fig. S2C-S2D).

## Discussion

The crucial importance of lipids in the malignant phenotype of PCa is clear [2]. The need for lipids by PCa cells can be met by taking up circulating lipids and enhancing de novo FA synthesis [15] or by lipid transfer from stromal adipocytes to PCa cells [16].

Recent studies have shown that FA can fuel prostate tumor growth [6], highlighting the importance of lipids in the progression of PCa. Selective degradation of intracellular lipid droplets by lipophagy as energy source has also been described in PCa by PPAT proximity [17].

In the present study, we found a specific PPAT lipid signature composed of 16 metabolites associated with PCa aggressiveness. Regression analysis showed that from these 16 metabolites, only MG (18:0) and a metabolite involved in the linoleic acid pathway (12–13-EpOME) were independent factors of PCa aggressiveness. However, we are aware that sample size may be one limitation and larger replication studies are needed in order to validate this panel.

The discovery of this signature raises the possibility that PPAT lipid profiling might provide independent information regarding PCa biology. By analyzing total FA composition (SFA, MUFA, and PUFA), we observed a significant increase only in MUFA and a significant decrease in ω-6 PUFA in high-risk PPAT samples. Few studies have investigated this issue in PPAT regarding PCa aggressiveness. While some authors found significantly higher ω-6 levels in PPAT when comparing patients with PCa or benign hyperplasia [7], others failed to observe any association between ω-6 in PPAT and PCa aggressiveness in Caucasians, whereas lower PPAT levels of ω-6 were protective in African-Caribbeans [8]. ω-6 FA are obtained from the diet as they cannot be synthesized in the human body. Thus far, human studies have failed to corroborate whether higher ω-6 levels are related to higher PCa risk or mortality [18]. By contrast, animal and in vitro studies have suggested that ω-6 PUFA stimulate, whereas ω-3 PUFA inhibit, PCa growth [19].

Our lipidomic PPAT analysis revealed significantly higher amounts of total LPI metabolites in samples from high-risk patients. LPI can be generated by the action of phospholipase A on lipids, and it has been established that it might play an important role in several diseases affecting various functions such as cell growth, differentiation, and motility in a number of cell types [20]. For example, the dysregulation of LPI in breast cancer has been related to migration properties [21]. Further investigations will be necessary to understand the role of LPI in PCa.

**Fig. 7:**
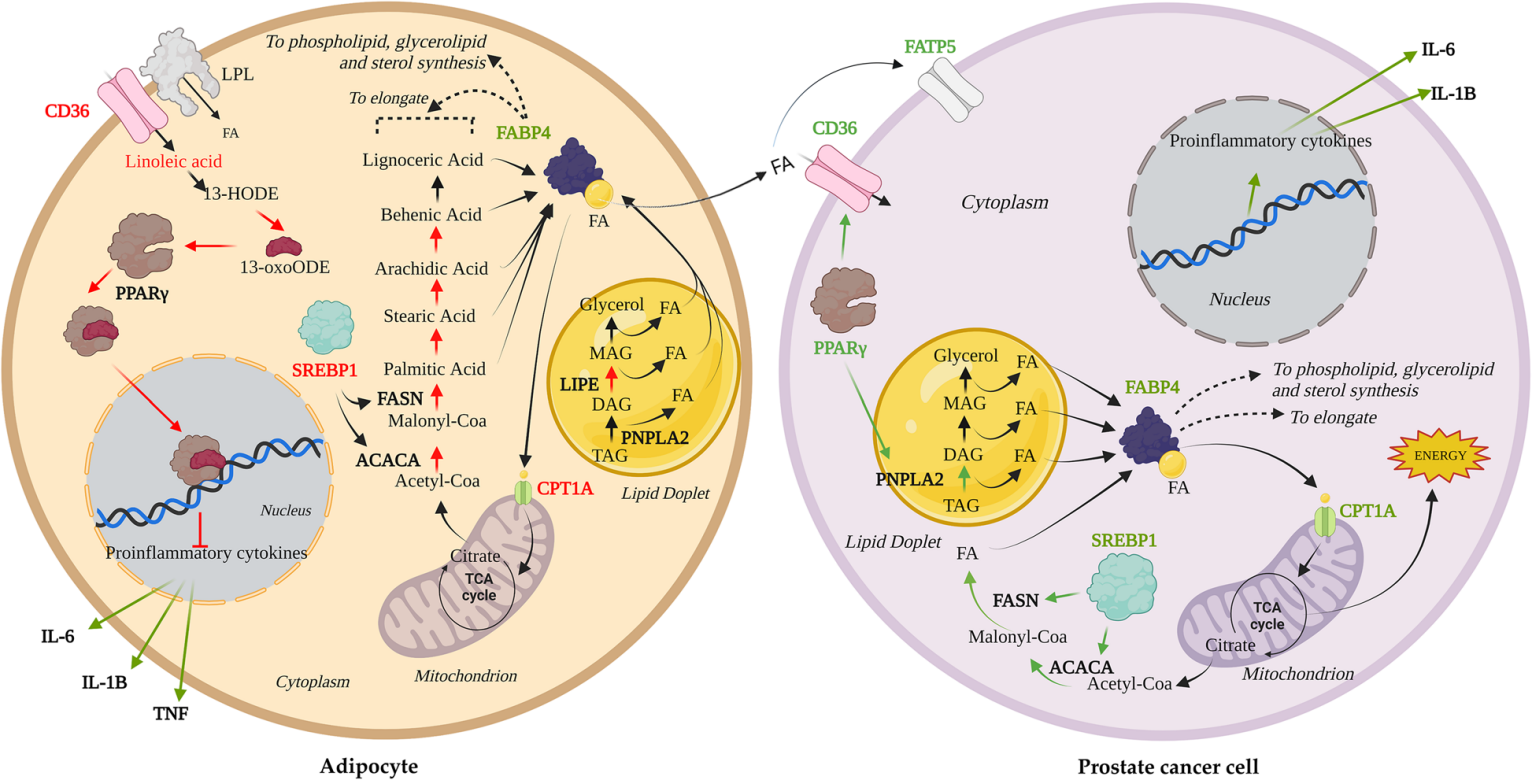
Scheme illustrating lipid metabolite alterations, related pathways, and implicated genes in human PPAT and PCa cell lines. In “aggressive” PPAT adipocytes, lower concentrations of 13-oxoODE, an endogenous ligand for PPARG, may provoke transcriptional upregulation of the proinflammatory cytokines *IL-6*, *IL-1B*, and *TNFα*. De novo lipogenesis is also reduced, as the expression levels of *ACACA* and *FASN* are downregulated. The resulting process translated to reduced concentrations of palmitic acid and its saturated FA intermediates (produced by elongation process): stearic acid, arachidic acid, behenic acid, and lignoceric acid. FAs from TG lipolysis can also be contemplated as another FA source. Reduced expression levels of lipase *LIPE* and the mitochondrial transporter *CPT1A* may indicate suppressed FA lipolysis. An increase in the expression of the *FABP4* lipid transporter gene points to active FA mobilization to PCa cells. PCa cells can take-up the FA released from PPAT adipocytes followed by an increased expression of lipid transporter genes (*CD36*, *FATP5*), fuelling cellular de novo lipogenesis and lipolysis. An active proinflammatory state is also observed in PCa cells. Abbreviations: 13-HODE, 13 hydroxy-octadecadienoic acid; 13-oxoODE, 13-oxo-octadecadienoic; PPARG, peroxisome proliferator-activated receptor gamma; IL-6, interleukin 6; IL-1B, interleukin 1 B; TNFα, tumor necrosis factor alpha; FASN, fatty acid synthase; SREBP-1, sterol regulatory element binding transcription factor 1; ACACA, acetyl-CoA; CPT1A, carboxylase alpha; Carnitine Palmitoyl transferase 1A; LIPE, hormone-sensitive lipase; MG, monoglyceride; DG, diglyceride; TG, triglyceride; FABP4, fatty acid binding protein 4; FA, fatty acid; PNPLA2, patatin-like phospholipase domain containing 2; CD36, CD36 molecule; LPL, lipoprotein lipase

Mapping of dysregulated FA metabolites onto metabolic pathways revealed a predominant involvement in de novo FA synthesis and linoleic acid metabolism (Fig. 7). Interestingly, all metabolites involved in these pathways were found to be lower in abundance in PPAT samples from high-risk PCa, clearly indicating a reduced lipid activity in the aggressive PCa-related fat. Interestingly we observed for the first time the reduced expression of genes implicated in de novo FA synthesis, namely *FASN*, *ACACA*, and *SREPB1* in high-risk PPAT (Fig. 7). The reduced expression of lipolytic-related genes such as *LIPE* was also observed in samples from high-risk patients, which was unexpected but nevertheless observed in co-cultured breast cancer cells with adipocytes [4], alluding to a unique adipocyte phenotype in this environment. Upregulation of inflammatory-related genes was also observed in the PPAT of patients with more aggressive PCa, indicating that PPAT is likely not inert.

To strengthen these findings, we performed co-culture experiments using PPAT explants and two PCa cell line models, which revealed ex vivo crosstalk between PPAT and PCa cells, with a dramatic reduction in lipid gene expression in PPAT explants accompanied by an activated inflammatory state characterized by overexpression of proinflammatory cytokines (Fig. 7). This reinforces the notion that adipocytes can provide lipids to tumor cells and participate in tumor aggressiveness [5]. By contrast, co-cultured PCa cell lines exhibited the opposite behavior with regard to lipid gene expression, which was clearly upregulated in PC-3 cells but did not change in LNCaP cells. Interestingly, the increased expression of fatty acid transporters such as *CD36* and *FATP5* was accompanied by an active uptake and accumulation of lipids in both cell lines when co-cultured with PPAT explants. In this sense, the incorporation of FFAs by PCa cells from neighboring adipocytes and similar uptake of exogenous lipids have been shown to occur in several PCa cell models [5, 22].

Proinflammatory-related genes (*IL-6*, *IL-1B*) were also overexpressed in PCa cell lines. The dysregulated inflammatory profile observed here, in both PCa cell lines and PPAT when in co-culture, especially in the case of *IL-6*, has been shown to play a key role in the acquired pro-invasive effect by tumor cells [4]. We also observed that both PCa cell lines gain aggressive properties when in contact with PPAT explants because expression of genes implicated in proliferation such as estrogen-related receptor alpha (*ESRRA*) [23] and matrix metallopeptidase 9 (*MMP-9*) [24] or involved in migration like twist family BHLH transcription factor 1 (*TWIST1*) [25] were clearly upregulated.

Although lipolysis was not evaluated in our ex vivo experiments, other authors showed that PPAT explants basal lipolysis was slightly increased in less aggressive PPAT PCa tissue compared to more aggressive ones, but did not reach significance [9]. Parameters related to PCa aggressiveness such as adipocyte size and stimulated FA release were not altered in their studied explant samples. Of note, when the authors evaluated explant basal FA release rates found a reduced tendency in high-risk PPAT explant samples when compared with the less aggressive counterparts, and only nervonic acid showed differences regarding aggressiveness [9]. In our PPAT analysis, nervonic acid levels were not altered between low- and high-degree PPAT samples, probably because we measured the total PPAT FA profile and not the secreted FA profile.

We also observed interesting changes in linoleic acid metabolism. Linoleic acid can be oxidized to produce 9- and 13-hydroxy-octadecadienoic acid (9- and 13-HODE) and 9- and 13-oxo-octadecadienoic acid (9- and 13-oxoODE). In human colonic epithelial cells, 13-oxoODE has been reported to be an endogenous ligand for PPARG with anti-inflammatory activity [14]. We found reduced concentrations of 13-oxoODE/9-oxoODE in high-risk PPAT samples and reduced expression levels of *PPARG*, a finding that could explain in part the reduced activity of this transcription factor (Fig. 7). Additionally, *ADIPOQ* gene, which is tightly regulated by *PPARG* [26], was found reduced in the aggressive PPAT samples. Although PPARG was initially identified for its role in adipocyte differentiation and in the regulation of genes involved in lipid and glucose metabolism [27], its activation can antagonize nuclear factor-kappa B (NF-ĸB) [28]. Thus, reduced expression of *PPARG* can also bolster the increased expression of proinflammatory cytokines observed in PPAT samples from aggressive PCa (Fig. 7). The increased expression of proinflammatory cytokines (*IL-6*, *TNFα*, *IL-1B*) found here in the aggressive PCa-related PPAT supports the role of PPAT in aggravating the tumor microenvironment, either by direct effects on premalignant cells or by acting on the tumor microenvironment [29].

Linoleic acid can also be converted by cytochrome P450 to epoxy-octadecenoic acids (EpOMEs) in the form of either 9(10)-EpOME (leukotoxin, coronaric acid) or its regioisomer 12(13)-EpOME (isoleukotoxin, vernolic acid). These leukotoxins can be produced by neutrophils, activating their chemotactic activity in disorders such as acute respiratory distress syndrome [30]. We detected lower levels of 12(13)-EpOME in high-risk PPAT; however, the physiological significance of EpOMEs in PCa needs to be further investigated.

To our knowledge, this is the first complete lipidomic profile analysis linked to biological activity regarding aggressiveness in PPAT. Accordingly, we believe that these data provide valuable information for future replication studies in larger cohorts.

## Conclusions

Overall, our findings show that the lipid composition of PPAT is altered in patients with high-risk PCa and is associated with changes in the PCa cell lipid metabolism affecting tumor cell processes.

## Supplementary Information

**Additional file 1: Table S1.** Clinical and anthropometrical characteristics of the patients.

**Additional file 2.** Additional Material and Methods.

**Additional file 3: Table S2.** Periprostatic adipose tissue lipidomic signatures.

**Additional file 4: Table S3.** Fatty acids in low-risk and high-risk PPAT.

**Additional file 5: Figure S1.** **A)** Partial least squares discriminant analysis (PLS-DA) model to evaluate the potential of the70 significant lipids to discriminate between low- and high-risk PPAT in2-dimensions and 3-dimensions. **B)** Variable importance in projection(VIP) showing the important metabolites that are discriminatory between low-risk PPAT and high-risk PPAT.

**Additional file 6: Figure S2.** **A)** Gene expression analysis of PPAT explants segregated according to aggressiveness after being co-cultured with PC-3 cells **B) **Gene expression analysis of PPAT explants segregated according toaggressiveness after being co-cultured with LNCaP cells **C)** Graph representing lipid uptake according to cell line and type of PPAT **D**)Graph representing lipid accumulation according to cell line and type of PPAT.

## Data Availability

The data underlying this article will be shared on reasonable request to the corresponding author.

## Abbreviations

12,13-EpOME: Vernolic acid
13OxoODE/9oxoODE: 13-Oxo-9,11-octadecadienoic acid/9-oxo-10E,12Z-octadecadienoic acid
ACACA: Acetyl-CoA carboxylase alpha
CD36: CD36 molecule
DG: Diglyceride
FA: Fatty acid
FABP4: Fatty acid binding protein 4
FAME: Fatty acid methyl ester
FASN: Fatty acid synthase
IL-1B: Interleukin 1 B
IL-6: Interleukin 6
LC–MS/MS: Liquid chromatography-mass spectrometry
LPC: Lysophosphatidylcholine
LPE: Lysophosphatidylethanolamine
LPI: Lysophosphatidylinositol
LPL: Lipoprotein lipase
MG: Monoglyceride
MUFA: Monounsaturated fatty acid
PC: Phosphatidylcholine (diacylglycerol)
PCa: Prostate cancer
PC-O: Phosphatidylcholine (alkyl-acyl-glycerol)
PLS-DA: Partial least squares-discriminant analysis score plot
PPARG: Peroxisome proliferator-activated receptor gamma
PPAT: Periprostatic adipose tissue
PUFA: Polyunsaturated fatty acid
SFA: Saturated fatty acid
SM: Sphingomyelin
SREBP-1: Sterol regulatory element binding transcription factor 1
TG: Triglyceride
TNFα: Tumor necrosis factor alpha
VIP: Variable importance in projection
