# Supplementary material for: The lipidomic profile of the tumoral periprostatic adipose tissue reveals alterations in tumor cell’s metabolic crosstalk
Source: BMC Med. 2022 Aug 18;20:255. doi: 10.1186/s12916-022-02457-3 (PMC9386931; doi:10.1186/s12916-022-02457-3)
Supplement: Supplementary file 1 — Additional file 1: Table S1. Clinical and anthropometrical characteristics of the patients. [file 12916_2022_2457_MOESM1_ESM.docx]

**Additional File 1**

|  | **ISUP GG Classification** | | | | | |  |  |  |
| --- | --- | --- | --- | --- | --- | --- | --- | --- | --- |
|  | **Low Risk** | | | **High Risk** | | |  |  |  |
|  | Group I (n=20)  Group II (n=0)  Total (n=20) | | | Group III (n=8)  Group IV (n=6)  Group V (n=6)  Total (n=20) | | |  |  |  |
|  | **Median (IQR)** | | | **Median (IQR)** | | | ***p*-value** | | |
| ***Anthropometric parameters*** | | | | | | | | | |
| **Age (years)** | 67.29 (61.25, 70.87) | | | 66.31 (63.00, 72.23) | | | 0.491 | | |
| **BMI (kg/m^2^)** | 26.54 (24.09, 28.80) | | | 25.47 (23.96, 28.51) | | | 0.931 | | |
| **Prostatic volume (cc)** | 54.80 (34.00, 84.25) | | | 42.1 (30.40, 52.75) | | | 0.165 | | |
|  |  |  |  |  |  |  |  |  |  |
| ***Glycaemic profile*** |  |  |  |  |  |  |  | | |
| **Glucose (mmol/L)** | 5.44 (4.79, 6.44) | | | 5.33 (4.44, 5.81) | | | 0.274 | | |
| **Insulin (pmol/L)** | 66.53 (51.92, 106.71) | | | 73.38 (59.21, 107.94) | | | 0.526 | | |
| **HOMA-IR** | 2.37 (1.64, 4.28) | | | 2,68 (1,97, 3.85) | | | 0.925 | | |
| **HbA1c (%)** | 5.50 (5.40, 6.30) | | | 5.40 (5.30, 5.78) | | | 0.183 | | |
|  |  |  |  |  |  |  |  |  |  |
| ***Lipid profile*** |  |  |  |  |  |  |  | | |
| **Cholesterol (mmol/L)** | 4.82 (4.19, 5.55) | | | 4.80 (4.21, 5.59) | | | 0.841 | | |
| **HDL cholesterol (mmol/L)** | 1.32 (1.14, 1.66) | | | 1.48 (1.11, 1.67) | | | 0.849 | | |
| **LDL cholesterol (mmol/L)** | 2.88 (2.24, 3.45) | | | 2.75 (2.40, 3.24) | | | 0.945 | | |
| **Triglycerides (mmol/L)** | 1.29 (0.92, 2.21) | | | 1.30 (0.90, 1.79) | | | 0.937 | | |
|  |  |  |  |  |  |  |  |  |  |
| ***Hepatic profile*** |  |  |  |  |  |  |  | | |
| **AST (µkat/L)** | 0.35 (0.27, 0.40) | | | 0.35 (0.32, 0.40) | | | 0.602 | | |
| **ALT (µkat/L)** | 0.33 (0.24, 0.48) | | | 0.34 (0.28, 0.42) | | | 0.395 | | |
| **GGT (µkat/L)** | 0.40 (0.30, 0.72) | | | 0.38 (0.29, 0.55) | | | 0.281 | | |
|  |  |  |  |  |  |  |  |  |  |
| ***Renal profile*** |  |  |  |  |  |  |  | | |
| **Uric acid (µmol/L)** | 370 (280, 445) | | | 360 (310, 405) | | | 0.428 | | |
| **Urea (mmol/L)** | 13.21 (10.89, 17.40) | | | 14.99 (11.87, 18.83) | | | 0.337 | | |
| **Creatinine (μmol/L)** | 79.56 (72.26, 94.37) | | | 84.42 (73.81, 100.97) | | | 0.830 | | |
|  |  |  |  |  |  |  |  |  |  |
| ***Hormonal profile*** |  |  |  |  |  |  |  | | |
| **SHBG (nmol/L)** | 41.50 (38.10, 54.35) | | | 39.05 (29.23, 44.76) | | | 0.204 | | |
| **Testosterone (nmol/L)** | 15.54 (12.36, 20.00) | | | 13.37 (10.23, 16.91) | | | 0.129 | | |
|  |  |  |  |  |  |  |  |  |  |
| ***Tumoral markers*** | | | | | | | | | |
| **Total PSA (μg/L)** | 5.59 (4.57, 7.08) | | | 7.41 (5.04, 14.61) | | | 0.010 | | |

**Additional Table S1. Clinical and anthropometrical characteristics of patient’s studied samples**

**Abbreviations: IQR:** Interquartile Range **ISUP (GG):** International Society of Urological Pathology Gleason Grading **BMI**, Body mass index; **cc**, centilitre; **HOMA-IR**, Homeostatic Model Assessment for Insulin Resistance; **HDL**, High-density lipoprotein; **LDL**, Low-density lipoprotein; **GGT**, Gamma Glutamyl transferase; **PSA**, Prostate specific antigen.
