# Supplementary material for: The lipidomic profile of the tumoral periprostatic adipose tissue reveals alterations in tumor cell’s metabolic crosstalk
Source: BMC Med. 2022 Aug 18;20:255. doi: 10.1186/s12916-022-02457-3 (PMC9386931; doi:10.1186/s12916-022-02457-3)
Supplement: Supplementary file 2 — Additional file 2. Additional Material and Methods. [file 12916_2022_2457_MOESM2_ESM.docx]

**Additional File 2**

**Additional Material and Methods**

**FAME ANALYSIS**

To obtain volatile fatty acids methyl ester derivatives (FAMEs), 10 mg of peri-prostatic adipose tissue samples were mixed with IS solution, chloroform and methanolic HCl and incubated at 80 ºC for 2 h. Afterwards, obtained FAMEs were extracted by a liquid-liquid extraction using hexane before to be injected on GC-MS system

FAME analysis by GC–MS was performed using an Agilent GC 7890A Series coupled to an Agilent QQQ 7000 Series (Agilent Technologies, Santa Clara, CA). Chromatographic separation was performed using a HP-88 (100 m x 250 μm x 0.25 μm) (Agilent). A volume of 1 µL of sample was automatically injected into a split/splitless inlet at a split rate of 20:1 and at a temperature of 240 °C. Helium (99.999% purity) was used as a carrier gas at a flow rate of 1 mL/min. To separate FAMEs an oven program between 140 and 240 ºC was applied. Ionisation was performed by electron impact (EI), with an electron energy of 70 eV and a source temperature of 250 °C.

**LIPIDOMICS (METHANOLIC EXTRACTION) ANALYSIS**

Extraction of lipidic methanolic fraction and was performed by adding 250 μL of methanol containing internal standards (Myristic acid-d27, Arachidonic acid-d8, Cholic acid-d4, taurocholic acid-d4, Lysophosphatidylcholine 18:1-d7 and Cortisol-d4) into a tube with approximately 20 mg of peri-prostatic adipose tissue. Then, the samples were homogenized on a Bullet Blender Homogenizer using Stainless-stell beads and incubated at -20ºC for 30 min., centrifuged at 15,000 rpm and supernatant was evaporated to dryness and reconstituted with 100 µL of methanol.

The separation of lipid species was performed by injection of 5 μl of samples into an UHPLC 1290 Infinity II (Agilent Technologies, Santa Clara, USA) equipped with an ACQUITY UPLC BEH C18 column (1.7 µm, 2.1 mm X 100 mm) (Waters Corporation, Mildford, MA, USA) thermostatized at 40 ºC. The elution was performed using water (A) and acetonitrile (B), containing both 0.05% formic acid, as mobile phases at a flow rate of 0.3 mL/min and using the following gradient program: 2 min, 50% B; 10 min, 98% B; 14.5 min, 98% B; and 15 min, 50% B. For the detection of lipids, the chromatograph was coupled to a qTOF 6550 series (Agilent Technologies) by an electrospray ionization source (ESI) operating in negative mode and the following parameters: gas temperature: 225 ºC; gas flow: 11 L/min; Nebulizer: 35 psi; sheath gas temperature: 300 ºC; sheath gas flow: 12 L/min; Capillary voltage: 3500 V; nozzle voltage: 500 V. The identification of lipid species was performed by matching their accurate mass and tandem mass spectrum, when available, to Metlin-PCDL from Agilent containing more than 40,000 metabolites and lipids. In addition, chromatographic behaviour of pure standards for each family and bibliographic information was used to ensure their putative identification.

**ACYLCARNITINES ANALYSIS**

The extraction of acylcarnitines was performed using the same procedure than in the lipidomics (methanolic extraction) analysis. In this case, methanol contains a mix of internal standard for acylcarnitines (C0 -D9, C2:0-D3, C3:0-D3, C4:0-D3, C5:0-D9, C8:0-D3, C14:0-D9 and C16:0-D9).

Acylcarnitines were analyzed using an UHPLC 1290 Infinity II coupled to a triple quadrupole mass spectrometer (QqQ-MS) by an ESI interface (Agilent Technologies). Chromatographic separation was performed using a Kinetex Polar C18 column (2.6 µm, 2.1 mm X 100 mm) (Phenomenex Inc., Torrance, CA, USA) by injecting 1 μL of sample. Mobile phases consist of water (A) and methanol (B), both with 0.1% formic acid at a flow rate of 0.4 mL/min and a gradient program as follow: 0 min, 0% B; 1 min, 0% B; 11 min, 100% B; 13 min, 100% B, 13.1 min, 0% B; 15 min, 0% B.

ESI interface and triple quadrupole mass spectrometer, working on positive mode and multiple reaction monitoring (MRM) mode, have set the following parameters: gas temperature: 200 ºC; gas flow: 6 L/min; Nebulizer: 30 psi; sheath gas temperature: 375 ºC; sheath gas flow: 11 L/min; Capillary voltage: 3500 V.

**LIPIDOMICS (FOLCH EXTRACTION) ANALYSIS**

A liquid-liquid extraction based on Folch procedure was performed by adding 750 μL of chloroform:methanol (2:1) containing internal standard (Lipidomic SPLASH®: lysophosphatidylcholine 18:1-d7, phosphatidylcholine 33:1-d7, sphingomyelin 36:2-d9, diacylglyceride 33:1-d7, triacylglyceride 48:1-d7 and cholesteroyl ester 18:1-d7) to approximately 10 mg of peri-prostatic adipose tissue. Then, the samples were homogenized on a Bullet Blender Homogenizer using Stainless-stell beads and incubated at -20ºC for 30 min. Afterwards, 240 µL of water with NaCl (0.8 %, w,v) was added and mixture was centrifuged at 15,000 rpm. Lower phase was recovered, evaporated to dryness, reconstituted with 400 μL of methanol:methyl-tert-butyl ether (9:1) and placed into chromatographic vials for their analysis.

Chromatographic separation was performed by injection of 2 μL of sample into an UHPLC 1290 Infinity II (Agilent Technologies) equipped with a KINETEX EVO C18 column (2.6 µm, 2.1 mm X 100 mm) (Phenomenex Inc.) thermostatized at 60 ºC. Elution program was consists in a ternary mobile phase containing water (A), methanol (B) and 2-propanol (C) with 10mM ammonium formate and 0.1% formic acid at a flow rate of 0.6 mL/min and a gradient as follow: 0 min, 10% B and 35% C; 0.5 min, 10 % B and 45% C; 1.5 min, 9.5% B and 47.7% C; 1.6 min, 7.5% B and 58.5% C; 5 min, 7% B and 61.2% C; 5.1 min, 4% B and 77.4% C; 7.5 min, 3.5% B and 80.1% C; 9 min, 3.5% B and 80.1% C; 9.5 min, 100% C; 11.5 min, 100% C; 11.6 min, 10% B and 45% C; 13min, 10% B and 45% C. For the detection of lipid species, chromatograph was coupled to a qTOF 6550 series (Agilent Technologies) by an electrospray ionization source (ESI) operating in positive mode and setting the following parameters: gas temperature: 225 ºC; gas flow: 11 L/min; Nebulizer: 35 psi; sheath gas temperature: 300 ºC; sheath gas flow: 12 L/min; Capillary voltage: 3500 V; nozzle voltage: 500 V. The identification of lipid species was performed by matching their accurate mass and tandem mass spectrum, when available, to Metlin-PCDL from Agilent containing more than 40,000 metabolites and lipids. In addition, chromatographic behaviour of pure standards for each family and bibliographic information was used to ensure their putative identification. The identification indicates the lipid family (PC-phosphatidylcholine, SM-sphingomyelin, DG-diacylglycerol, TG-triacylglycerol and MG-monoacylglycerol), the total number of carbons of the acyl chains and the number of double bonds.
