# Supplementary material for: The lipidomic profile of the tumoral periprostatic adipose tissue reveals alterations in tumor cell’s metabolic crosstalk
Source: BMC Med. 2022 Aug 18;20:255. doi: 10.1186/s12916-022-02457-3 (PMC9386931; doi:10.1186/s12916-022-02457-3)
Supplement: Supplementary file 3 — Additional file 3: Table S2. Periprostatic adipose tissue lipidomic signatures. [file 12916_2022_2457_MOESM3_ESM.docx]

**Additional File 3**

| Lipid species | Names | | | Low Risk (n= 20) Median ± [range] | High Risk (n= 20) Median ± [range] | | |
| --- | --- | --- | --- | --- | --- | --- | --- |
|  |  |  |  |  |  |  |  |
| Fatty acids (FAME) |  | | |  | |  |  |
|  |  |  |  |  |  |  |  |
| FA(14:0) (μmol/mg) | Myristic Acid | | | 38.64 [32.77; 49.92] | 31.36 [27.72; 41.37] | | |
| FA(16:0) (μmol/mg) | Palmitic Acid | | | 238.52 [207.70; 265.10] | 215.38 [187.26; 243.61] | | |
| FA(18:0) (μmol/mg) | Stearic Acid | | | 64.34 [53.23; 75.35] | 53.02 [47.04; 66.19] | | |
| FA(20:0) (μmol/mg) | Arachidic Acid | | | 3.38 [2.12; 4.18] | 2.36 [2.14; 3.04] | | |
| FA(22:0) (μmol/mg) | Behenic Acid | | | 0.60 [0.41; 0.81] | 0.40 [0.31; 0.51] | | |
| FA(23:0) (μmol/mg) | Tricosanoic Acid | | | 0.04 [0.03; 0.06] | 0.03 [0.02; 0.04] | | |
| FA(24:0) (μmol/mg) | Lignoceric Acid | | | 0.26 [0.16; 0.42] | 0.19 [0.14; 0.23] | | |
| FA(18:2n-6) | Linoleic Acid | | | 247.25[180.58;312.61] | 182.29 [144.40; 228.12] | | |
| LIP-I (more hydrophobic lipids) |  | | |  | |  |  |
| FA(12:0) | Lauric Acid | | | 1.30 [1.16; 1.64] | 1.19 [0.99; 1.32] | | |
| FA(14:0) | Myristic Acid | | | 3.98 [3.23; 5.16] | 3.15 [2.75; 4.75] | | |
| FA(15:0) | Pentadecanoic Acid | | | 1.07 [0.88; 1.26] | 0.86 [0.69; 1.15] | | |
| FA(18:0) | Stearic Acid | | | 37.91 [31.87; 45.45] | 33.13 [24.33; 42.07] | | |
| 12,13-EpOME | Vernolic Acid | | | 0.61 [0.46; 1.41] | 0.44 [0.34; 0.63] | | |
| 9,12,13-TriHOME | Pinellic acid | | | 2.80 [2.31; 3.27] | 3.31 [2.48; 3.83] | | |
| FA(18:2n-6 (iso-2)) | Linoleic Acid iso-2 | | | 1.19 [0.95; 2.08] | 0.96 [0.60; 1.32] | | |
| FA(18:2n-6 (iso-1)) | Linoleic Acid | | | 39.43 [32.05; 59.94] | 31.57 [24.98; 47.68] | | |
| FA(21:2n-7) | 11,13-Eicosadienoic Acid | | | 3.69 [2.59; 4.17] | 2.70 [1.59; 3.48] | | |
| 15,16-EpODE | 15,16-epoxy-13-OH-9Z,11E-octadecadienoic acid | | | 0.75 [0.63; 0.96] | 0.60 [0.53; 0.83] | | |
| 13-oxoODE/9-oxoODE | 13-Oxo-9,11-octadecadienoic acid/9-oxo-10E,12Z-octadecadienoic acid | | | 0.60 [0.29; 1.11] | 0.27 [0.13; 0.55] | | |
| LPI(18:2) | 1-(9Z,12Z-octadecadienoyl)-glycero-3-phospho-(1'-myo-inositol) | | | 0.25 [0.16; 0.41] | 0.36 [0.27; 0.51] | | |
| LPI(16:0) | 1-hexadecanoyl-sn-glycero-3-phospho-(1'-myo-inositol) | | | 0.52 [0.42; 0.81] | 0.84 [0.60; 1.10] | | |
| LPI(18:1) | 1-(9Z-octadecenoyl)-sn-glycero-3-phospho-(1'-myo-inositol) | | | 2.08 [1.11; 3.52] | 2.97 [1.93; 4.83] | | |
| LPI(18:0) | 1-octadecanoyl-sn-glycero-3-phospho-(1'-myo-inositol) | | | 7.12 [5.25; 10.17] | 9.51 [6.04; 14.02] | | |
| LPE(20:5)sn2 | 2-(5Z,8Z,11Z,14Z,17Z-eicosapentaenoyl)-sn-glycero-3-phosphoethanolamine | | | 0.18 [0.15; 0.25] | 0.31 [0.24; 0.48] | | |
| LPE(20:5)sn1 | 1-(5Z,8Z,11Z,14Z,17Z-eicosapentaenoyl)-glycero-3-phosphoethanolamine | | | 0.05 [0.03; 0.08] | 0.09 [0.06; 0.13] | | |
| LPE(22:6)sn2 | 2-(4Z,7Z,10Z,13Z,16Z,19Z-docosahexaenoyl)-sn-glycero-3-phosphoethanolamine | | | 0.51 [0.41; 0.73] | 0.69 [0.50; 1.05] | | |
| LPE(18:2)sn2 | 2-(9Z,12Z-octadecadienoyl)-sn-glycero-3-phosphoethanolamine | | | 10.39 [8.34; 12.60] | 7.94 [6.16; 10.33] | | |
| LPE(20:4)sn2 | 2-(5Z,8Z,11Z,14Z-eicosatetraenoyl)-sn-glycero-3-phosphoethanolamine/2-(8Z,11Z,14Z,17Z-eicosatetraenoyl)-sn-glycero-3-phosphoethanolamine | | | 6.57 [5.45; 8.06] | 8.35 [6.86; 10.40] | | |
| LPE(20:2)sn1 | 1-(11Z,14Z-eicosadienoyl)-glycero-3-phosphoethanolamine | | | 0.10 [0.07; 0.11] | 0.07 [0.06; 0.09] | | |
| LPC(14:2)sn2 | 2-tetradecanoyl-sn-glycero-3-phosphocholine | | | 0.02 [0.02; 0.03] | 0.03 [0.02; 0.04] | | |
| LPC(20:5)sn2 | 2-[(5Z,8Z,11Z,14Z,17Z)-eicosapentaenoyl]-sn-glycero-3-phosphocholine | | | 0.02 [0.01; 0.03] | 0.03 [0.02; 0.04] | | |
| LPC(16:1)sn2 | 2-(9Z-hexadecenoyl)-sn-glycero-3-phosphocholine /2-(9E-hexadecenoyl)-sn-glycero-3-phosphocholine | | | 0.08 [0.05; 0.10] | 0.12 [0.08; 0.15] | | |
| LPC(22:6)sn2 | 2-(4Z,7Z,10Z,13Z,16Z,19Z-docosahexaenoyl)-sn-glycero-3-phosphocholine | | | 0.02 [0.01; 0.03] | 0.04 [0.02; 0.06] | | |
| LPC(20:4)sn2 | 2-(5Z,8Z,11Z,14Z-eicosatetraenoyl)-sn-glycero-3-phosphocholine | | | 0.32 [0.24; 0.44] | 0.52 [0.33; 0.71] | | |
| LPC(17:1)sn2 | 1-(10Z-heptadecenoyl)-sn-glycero-3-phosphocholine | | | 0.004 [0.001; 0.008] | 0.01 [0.003; 0.014] | | |
| LPC(20:3)sn2 | 1-(5Z,8Z,11Z-eicosatrienoyl)-sn-glycero-3-phosphocholine | | | 0.07 [0.06; 0.10] | 0.10 [0.08; 0.14] | | |
| LIP-II (less hydrophobic lipids) |  | | |  | |  |  |
| MG(18:0) |  | | | 67.56 [54.22; 104.91] | 48.27 [42.88; 61.48] | | |
| DG(34:0) |  | | | 16.07 [14.01; 20.84] | 16.87 [14.3; 18.71] | | |
| TG(48:4) |  | | | 33.97 [19.44; 45.07] | 25.68 [18.64; 27.98] | | |
| TG(50:4) |  | | | 313.38 [199.07; 395.65] | 230.41 [185.47; 263.82] | | |
| TG(52:5) |  | | | 524.37 [340.23; 643.99] | 340.26 [292.80; 403.39] | | |
| TG(51:4) |  | | | 38.49 [32.20; 57.51] | 32.56 [24.03; 36.19] | | |
| TG(54:6) |  | | | 773.70 [436.19; 1320.21] | 389.25 [285.82; 586.69] | | |
| TG(53:5) |  | | | 31.15 [23.53; 36.74] | 22.93 [18.27; 27.94] | | |
| TG(52:4) |  | | | 7513.8 [4966.69; 8971.21] | 4940.05 [4089.82; 5815.22] | | |
| TG(51:3) |  | | | 255.97 [245.70; 356.63] | 216.98 [183.35; 271.24] | | |
| TG(54:5) |  | | | 7235.8 [4216.47; 9657.85] | 4349.93 [3405.53; 5566.82] | | |
| TG(53:4) |  | | | 181.51 [149.13; 253.33] | 157.29 [128.31; 172.50] | | |
| TG(52:3) |  | | | 25251. 2 [22296.2; 28698] | 22346.2 [20511.67; 24516.2 | | |
| TG(55:4) |  | | | 68.29 [55.86; 74.15] | 56.13 [46.18; 69.40] | | |
| TG(52:0) |  | | | 20.31 [15.12; 25.58] | 16.77 [12.21; 19.78] | | |
| TG(54:1) |  | | | 267.32 [144.27; 333.93] | 190.62 [145.93; 232.44] | | |
| TG(60:5) |  | | | 5.78 [4.16; 6.99] | 4.43 [2.30; 5.97] | | |
| TG(58:3) |  | | | 63.12 [49.75; 79.89] | 47.31 [33.58; 71.95] | | |
| TG(55:1) |  | | | 7.16 [4.59; 9.65] | 5.47 [4.10; 6.61] | | |
| TG(60:4) |  | | | 13.76 [9.58; 17.62] | 9.30 [7.12; 11.37] | | |
| TG(56:1) |  | | | 39.56 [35.51; 51.51] | 27.50 [17.35; 34.78] | | |
| TG(58:2) |  | | | 59.51 [44.43; 72.08] | 38.46 [23.56; 51.60] | | |
| TG(60:3) |  | | | 32.78 [22.18; 42.91] | 22.16 [14.67; 25.54] | | |
| TG(58:1) |  | | | 30.27 [23.24; 35.60] | 18.35 [11.68; 26.14] | | |
| TG(60:2) |  | | | 36.16 [23.92; 45.44] | 22.31 [14.16; 30.01] | | |
| SM(18:1;2/20:0) |  | | | 1.19 [0.90; 1.52] | 1.22 [0.97; 1.39] | | |
| PC(32:0) |  | | | 0.54 [0.43; 0.72] | 0.64 [0.56; 0.79] | | |
| PC-O(36:5) |  | | | 0.11 [0.09; 0.17] | 0.19 [0.15; 0.28] | | |
| PC-O(36:4) |  | | | 0.21 [0.19; 0.29] | 0.27 [0.22; 0.32] | | |
| PC-O(38:6) |  | | | 0.03 [0.02; 0.04] | 0.06 [0.05; 0.08] | | |
| Acylcarnitines | |  |  | | |  |  |
| C3DC | Malonyl carnitine | | | 1.14 [0.89; 1.48] | 1.29 [1.06; 1.54] | | |

**Additional Table S2.** **Periprostatic adipose tissue lipidomic signatures.** Significant signatures after comparing Low PCa PATT vs High PCa PATT. Abbreviations: FA: Fatty acid; LPI: lysophosphatidylinositol: LPE: lysophosphatidylethanolamine; LPC: lysophosphatidylcholine; MG: monoglyceride; DG: diglyceride; TG: triglyceride; PC: phosphatidylcholine (diacylglycerol); PC-O: phosphatidylcholine (alkyl-acyl-glycerol); SM: sphingomyelin
