# Supplementary material for: The lipidomic profile of the tumoral periprostatic adipose tissue reveals alterations in tumor cell’s metabolic crosstalk
Source: BMC Med. 2022 Aug 18;20:255. doi: 10.1186/s12916-022-02457-3 (PMC9386931; doi:10.1186/s12916-022-02457-3)
Supplement: Supplementary file 4 — Additional file 4: Table S3. Fatty acids in low-risk and high-risk PPAT. [file 12916_2022_2457_MOESM4_ESM.docx]

| **Fatty Acids (FA)** | **Low Risk** | | **High Risk** | |
| --- | --- | --- | --- | --- |
|  | **Median (Range)** | **% over Total [Median (Range)]** | **Median (Range)** | **% over Total [Median (Range)]** |
| **Saturated Fatty Acids (SFA)** | 355.69 (314.26, 422.93) | 28.53 (26.24, 31.22) | 320.46 (273.91, 366.72) | 27.59 (26.55, 30.11) |
| Dodecanoic acid (C12:0) | 12.99 (5.52, 17.42) |  | 9.23 (4.55, 15.08) |  |
| Myristic acid (C14:0) | 38.64 (32.77, 49.91) |  | 31.36 (27.71, 41.37) |  |
| Pentadecanoic acid (C15:0) | 3.72 (3.12, 4.64) |  | 3.46 (2.64, 4.39) |  |
| Palmitic acid (C16:0) | 238.52 (207.69, 265.10) |  | 215.38 (187.26, 243.61) |  |
| Heptadecanoic acid (C17:0) | 3.67 (3.24, 4.58) |  | 3.42 (3.06, 3.93) |  |
| Stearic acid (C18:0) | 64.34 (53.23, 75.35) |  | 53.02 (47.04, 66.19) |  |
| Arachidic acid (C20:0) | 3.38 (2.11, 4.18) |  | 2.35 (2.14, 3.04) |  |
| Behenic acid (C22:0) | 0.59 (0.41, 0.81) |  | 0.39 (0.31, 0.51) |  |
| Tricosanoic acid (C23:0) | 0.04 (0.03, 0.06) |  | 0.03 (0.02, 0.04) |  |
| Lignoceric acid (C24:0) | 0.26 (0.16, 0.42) |  | 0.19 (0.14, 0.23) |  |
|  |  |  |  |  |
| **Monounsaturated Fatty Acid (MUFA)** | 663.69 (545.41, 686.03) | 49.99 (48.23, 52.33) | 622.13 (552.44, 675.15) | 54.68 (50.11, 56.59) |
| Myristoleic acid (C14:1 [cis-9]) | 2.89 (2.25, 3.81) |  | 2.60 (2.04, 3.24) |  |
| Palmitoleic acid (C16:1 [cis-9]) | 35.40 (26.81, 51.83) |  | 34.27 (28.48, 47.20) |  |
| Oleic acid (C18:1 [cis-9]) | 620.48 (505.07, 651.01) |  | 578.08 (524.79, 627.38) |  |
| Erucic acid (C22:1 [cis-13]) | 0.38 (0.31, 0.52) |  | 0.36 (0.25, 0.52) |  |
| Nervonic acid (C24:1 [cis-15]) | 0.22 (0.19, 0.29) |  | 0.20 (0.14, 0.30) |  |
|  |  |  |  |  |
| **Omega-3 (ω-3) polyunsaturated fatty acids (PUFA)** | 9.62 (8.33, 12.61) | 0.79 (0.64, 1.06) | 9.98 (8.29, 11.82) | 0.82 (0.73, 1.09) |
| Linolenic acid (C18:3 [cis-9,12,15]) | 7.21 (5.80, 8.02) |  | 6.32 (5.19, 8.35) |  |
| Eicosapentanoic acid (C20:5 [cis-5,8,11,14,17]) | 0.55 (0.38, 0.79) |  | 0.67 (0.42, 1.00) |  |
| Docosahexanoic acid (C22:6 [cis-4,7,10,13,16,19]) | 2.20 (1.95, 3.03) |  | 2.66 (1.97, 3.69) |  |
|  |  |  |  |  |
| **Omega-6 (ω-6) polyunsaturated fatty acids (PUFA)** | 262.78 (190.63, 326.64) | 18.98 (16.31, 22.78) | 194.83 (153.97, 239.66) | 17.05 (13.76, 18.52) |
| Linoleic acid (C18:2 [cis-9,12]) | 247.25 (180.58, 312.61) |  | 182.29 (144.40, 228.12) |  |
| Linolenic acid (C18:3 [cis-6,9,12]) | 1.17 (0.74, 1.46) |  | 0.86 (0.71, 1.25) |  |
| Eicosadienoic acid (C20:2 [cis-11,14]) | 4.43 (3.73, 5.55) |  | 3.85 (3.18, 4.26) |  |
| Arachidonic acid (C20:4 [cis-5,8,11,14]) | 6.22 (5.49, 8.56) |  | 6.62 (5.87, 8.92) |  |
